# Suicidal behavior and spiritual functioning in a sample of Veterans diagnosed with PTSD

**DOI:** 10.5249/jivr.v8i1.728

**Published:** 2016-01

**Authors:** Marek S. Kopacz, Joseph M. Currier, Kent D. Drescher, Wilfred R. Pigeon

**Affiliations:** ^*a*^US Department of Veterans Affairs, VISN 2 Center of Excellence for Suicide Prevention, Canandaigua, NY.; ^*b*^Psychology Department, University of South Alabama, Mobile, AL.; ^*c*^US Department of Veterans Affairs, National Center for PTSD: Dissemination & Training Division, Menlo Park, CA.; ^*d*^Department of Psychiatry, University of Rochester Medical Center, Rochester, NY.

**Keywords:** Spirituality, Veterans, Suicide, Post-traumatic stress disorder

## Abstract

**Background:**

Spiritual well-being has been lauded to exert a protective effect against suicidal behavior. This study examines the characteristics of spiritual functioning and their association with a self-reported history of suicidal thoughts and behavior in a sample of Veterans being treated for post-traumatic stress disorder (PTSD).

**Methods:**

The sample includes 472 Veterans admitted to a PTSD Residential Rehabilitation Program. Measures included the Brief Multidimensional Measure of Religiousness and Spirituality, PTSD Checklist – Military Version, Combat Experiences Scale, and individual items pertaining to history of suicidal thoughts and attempts, spiritual practices, and select demographics.

**Results:**

Problems with forgiveness and negative religious coping were uniquely associated with suicide risk, above and beyond age, gender, or ethnicity, combat exposure, and severity of PTSD symptomatology. Organizational religiousness was associated with decreased risk for thinking about suicide in the presence of these covariates. Daily spiritual experiences were inversely associated with suicidal thoughts. Differences in spirituality factors did not distinguish Veterans with both suicidal ideation and prior attempts from those who had ideations absent any prior attempts.

**Conclusions:**

The findings suggest that enhanced or diminished spiritual functioning is associated with suicidal thoughts and attempts among Veterans dealing with PTSD.

## Introduction

In recent years, preventing suicidal behavior among former military personnel has risen to that of a major public health challenge. The number of Veterans who die by suicide is estimated at 18-22 per day.^1^ Also, a disproportionally high percentage of suicide deaths in the United States (US) – approximately 18% – are attributed to current and former military personnel.^2^ In an effort to combat this alarming trend, the US Department of Veterans Affairs (VA) began a concentrated effort aimed at preventing suicidal behavior in Veteran populations. ^3,4^ This includes a targeted focus on providing supportive services to select Veteran populations recognized as being at high-risk of suicide. 

Veterans diagnosed with or exhibiting symptoms of post-traumatic stress disorder (PTSD) represent a population at increased risk of suicide.^5-9^ In FY2011, VA Medical Centers (VAMCs) provided healthcare services to 476,515 Veterans for complaints related to PTSD.^10^ Whereas the lifetime prevalence of PTSD in the general population is estimated at 7.8%,^11^ the prevalence of this disorder is estimated at 12.1% in Veterans of Operation Desert Storm,^12^ 13.8% for Veterans of Operations Iraqi Freedom and Enduring Freedom,^13^ and 15.2% (men)/8.5% (women) for those who served during the Vietnam era. ^14,15^

An often overlooked dimension of both suicide risk and treatment for at-risk populations is how spiritual functioning might influence health outcomes.^16^

A recent position article argues for the utility of applying a sense of spiritual well-being, founded on common principles and a shared common experience of spirituality, to suicide prevention efforts.^17^ While considerable debate exists as to the extent to which spirituality and religion represent similar as opposed to distinct constructs,^18^ general consensus has been reached as to the common religious and spiritual domains applicable to health outcomes research.^19^ In keeping with the unique multidimensionality of spiritual functioning, these domains include, among others, daily spiritual experiences, forgiveness, private religious practices, religious coping, and organizational religiousness. ^19^

A growing body of research continues to find significant associations linking select domains of spiritual functioning with positive and negative health outcomes associated with trauma. For example, positive forms of religious coping can be used to adaptively deal with problematic situations, inclusive of any ensuing emotional unrest.^20-23^ In contrast, negative religious coping and problems with forgiveness have been implicated in increasing one’s risk for developing PTSD. ^24^ One study also found that select measures of spiritual functioning can be used to predict later PTSD symptom severity in Veterans. ^25^

Spiritual functioning could also impact help-seeking behaviors among Veterans dealing with PTSD. Research suggests that some Veterans will actively seek-out healthcare services motivated more by guilt and weakened religious faith, as opposed to the severity of their PTSD symptoms. ^26^ Traumatic experiences are also thought to contribute to a desire for personal growth and meaning-making.^27^ This search for meaning appears to reinforce the continued involvement in mental health treatment of Veterans dealing with PTSD.^28^ It seems reasonable to assume that many treatment-seeking Veterans might then feel a need to address existential and possible spiritual concerns as part of their recovery process.

Available literature suggests that select dimensions of spiritual functioning are not foreign to many Veterans. For example, Veterans are known to ascribe varying degrees of importance to religion and spirituality.^29-31^ The number of Veterans who report attending religious services at least once per month is currently estimated at 49%.^32^ Veterans are also known to actively engage in religious and spiritual coping practices.^33-35^ Some suggest that enhanced spiritual functioning also appears to be relevant to Veteran suicide prevention efforts. In one study, Veterans with a history of suicide ideation self-rated their spiritual health as worse than that of Veterans without a history of suicide ideation. ^36^

The aim of this study is to examine the characteristics of spiritual functioning which may be associated with differences in self-reported history of suicidal thoughts and behavior in a sample of Veterans being treated for PTSD. While a sense of spiritual and/or religious well-being is lauded as protective against suicide,^37-40^ no published data exists to document the validity of this relationship in an at-risk population of Veterans. We hypothesized that Veterans who reported higher levels of adaptive spiritual functioning would have a lower prevalence of suicidal thoughts/behaviors compared to Veterans with lower levels of spiritual functioning. 

## Methods

**Participants and Procedure **

Data were collected among patients admitted for treatment to one of two VA PTSD Residential Rehabilitation Programs (PRRPs) which provide treatment to Veterans from all service eras, consisting of a 45-bed program for men and 10-bed program for women. The PRRP lasts from 60 to 90 days. Admissions to the programs were based on clinician referrals for Veterans with severe PTSD who had not improved sufficiently through less intensive treatment options. In cases where Veterans had more than one admission to the PRRP during the study period, we only incorporated information from their first admission. Exclusion criteria for the PRRPs included psychotic symptoms, alcohol/drug misuse within the previous 14 days, and medical conditions that would interfere with engagement in treatment activities/procedures. 

Participants each had a current diagnosis of PTSD made by a VA health care provider on the basis of clinical interviews and supported by commonly used assessment instruments. Diagnostic information for other psychiatric disorders was not available for most of the sample. No data was also available to describe the clinical history of the sample related to their diagnosis of PTSD (e.g., variability in symptom severity over time, length of time since diagnosis). Be that as it may, this sample was predominantly comprised of Vietnam Veterans. Only 9.7% of the sample included individuals who served in Iraq or Afghanistan. Consequently one can reasonably assume that the sample population principally included individuals with a more chronic course of PTSD. 

All measures that formed the basis of this study were completed primarily for clinical decision-making and quality management of the PRRPs. Prior to the collection of data, however, a consent process was approved by Stanford University’s Institutional Review Board (IRB) for Human Subjects in Medical Research and the VA Research and Development (R&D) Committee at the VA Palo Alto Health Care System that allowed these Veterans to provide written permission on the pre-treatment questionnaire for their clinical assessments to be used for research purposes. In 2007, this protocol was closed and a de-identified data set was respectively approved by the Stanford IRB and VA R&D Committees for the types of analyses that form the basis of this study.

The sample population for this study initially included 807 Veterans who sought treatment at the PRRP during the period 2002-2007.^41^ Data were initially collected at the start of the PRRP for treatment planning and evaluation purposes. The final sample for the present analysis includes 472 Veterans who completed assessments of both spirituality and history of suicidal thoughts/behaviors at their intake assessment. 

**Measures**

The Brief Multidimensional Measure of Religiousness and Spirituality (BMMRS) is a 40-item scale developed to assess twelve dimensions of spirituality in behavioral health research with religiously heterogeneous groups.^19,42^ Items for the individual subscales were coded such that lower scores indicate higher levels of each construct. Chronbach’s alpha for this instrument ranges from .91 to .95 across different samples.^19^

We used a 6-item version of the Daily Spiritual Experiences Scale for assessing ordinary, day-to-day experiences of spirituality (e.g., feel presence of higher power, sense of peace or harmony, touched by beauty of universe).^43^ Items are scored on a 6-point Likert scale (1-6) with scores ranging from 6 to 36. High scores on the scale are indicative of an infrequent occurrence of day-to-day spiritual experiences. This scale has been consistently found to have a Chronbach’s alpha higher than .89.^44^

Forgiveness was measured with three questions pertaining to self-forgiveness, interpersonal forgiveness, and forgiveness from God.^45^ Items are scored on a 4-point Likert scale ranging from 1 (Always or almost always) to 4 (Never). High scores on the scale are indicative of an infrequent experience of forgiveness.

Private spiritual practices were examined with items assessing engagement in prayer, meditation, reading religious material, and listening to religious programming. Scoring is on an eight-point Likert scale ranging from 1 (More than once a day) to 8 (Never). High scores on the scale are indicative of an infrequent engagement in private spiritual practices.

We also incorporated scales for assessing positive and negative forms of religious coping using items derived from the RCOPE.^46,47^ Scoring for each 3-item scale utilized a 4-point Likert scale ranging from 1 (A great deal) to 4 (Not at all). High scores on the scale are indicative of a low degree of positive or negative religious coping, respectively.

Organizational religiousness was measured with two items assessing involvement in a church or other formal religious group.^48^ Responses are on an eight-point Likert scale ranging from 1 (More than once a day) to 8 (Never). High scores on the scale are indicative of infrequent involvement in formal religious activities.

Two questions were incorporated in the clinical intake questionnaire for assessing Veterans’ history of suicidal thoughts and prior attempts. First, suicide ideation was assessed with the question: "Have you ever had serious thoughts of committing suicide?" Veterans were then asked, "Have you attempted suicide in your lifetime?" Responses to these questions were each scored in a "yes" or "no" format. Drawing on these items, a single three-category variable was developed for the analysis that gauged Veterans’ risk status for suicide: No Suicide (i.e., "no" on both items), Ideation Only (i.e., "yes" on ideation item and "no" on attempt item), and Ideation/Attempt (e.g., "yes" on both items). No patients checked "yes" on the attempt item and "no" on the ideation item.

The PTSD Checklist – Military Version (PCL-M) a 17-item self-report instrument assessing severity of PTSD symptoms over the past month.^49,50^ Items are rated on a five-point scale, with anchor points of 1 (not at all) to 5 (extremely). The total score on the PCL-M can range from 17 to 85. Given the link between spirituality factors and PTSD, Veterans’ total scores on this measure were incorporated as a covariate in statistical analyses. Chronbach’s alpha for this instrument range from .94 to .97.^49,51^

The Combat Experiences Scale (CES) is a well-established 7-item measure.^52^ Some items are rated on 4 or 5 point frequency (1 = "no" or "never" to 5 = "more than 50 times") or (1 = "no" to 4 = "more than 12 times"), duration (1 = "never" to 5 = "more than 6 months"), or degree of loss (1 = "no one" to 4 = "more than 50%") scale. Total scores range from 0 to a maximum of 41 with higher scores indicating greater exposure to combat stressors. Given varying rates of combat exposure in the sample, the CES was also included as a covariate in the analyses. One study placed Chronbach’s alpha for the CES at .93.^53^

Demographic variables analyzed as part of this study included age, gender, ethnicity, marital status, and religious affiliation.

**Statistical analysis**

Differences in mean (SD) age as well as mean (SD) scores for the CES and PCL-M, respectively, were analyzed across the three suicide risk groups using analysis of variance (ANOVA). Differences in the frequency distribution of the remaining demographic variables were analyzed using chi-squared (χ2) tests. 

Multivariate analysis of covariance (MANCOVA) was used to examine the relationship between suicide risk and spirituality.^54^ Analysis of covariance (ANCOVA) was next applied for a step-down analysis.^55^ ANCOVA is considered most appropriate when controlling for individual attributes and is oftentimes applied in non-experimental analyses without randomized sample populations.^55,56^ Lastly, Fisher’s Least Significant Difference (LSD) was run for comparisons based on the results of ANCOVA.^56^ Fisher’s LSD allows for identifying significant relationships while controlling for family wise error.^57^ For the purposes of this study, suicide risk (i.e., No Suicide, Ideation Only, and Ideation/Attempt) is treated as the dependent variable. The remaining measures used in this study are treated as covariates.

## Results

No differences were noted across the three groups for age, ethnicity, religious affiliation, or PCL-M score (Table 1). Differences were noted only for three variables. Compared to the No Suicide and Ideation Only groups, there appeared to be more females in the Ideation/Attempt group (χ2(2) = 7.72, p = .02). The greatest percentage of cases who reported being separated from their spouse/partner was noted in the Ideation/Attempt group, followed by the No Suicide, and Ideation Only group (χ2(2) = 7.52, p = .02). Lastly, the highest CES scores were noted in the No Suicide group, followed by the Ideation Only group, and the Ideation/Attempt group, which had the lowest mean CES scores (F(2, 469) = 7.87, p < .001). 

**Table 1:** Demographic characteristics by history of suicide (n=472).

	No Suicide (n = 136) N (%)	Ideation Only (n = 165) N (%)	Ideation/Attempt (n = 171) N (%)
Age [M (SD)]	51.54 (11.61)	50.55 (9.86)	50.66 (10.57)
Gender *			
Male	131 (96.3%)	159 (96.4%)	154 (90.1%)
Female	5 (3.7%)	6 (3.6%)	17 (9.9%)
**Ethnicity**			
Caucasian	77 (56.6%)	105 (63.6%)	97 (56.7%)
African American	25 (18.4%)	26 (15.8%)	26 (15.2%)
Hispanic	21 (15.4%)	24 (14.5%)	28 (16.4%)
Other minority ^a^	13 (9.6%)	10 (6.1%)	20 (11.7%)
**Marital Status**			
Married	31 (22.8%)	34 (20.6%)	41 (24.0%)
Separated *	12 (8.8%)	8 (4.8%)	23 (13.5%)
Divorced	45 (33.1%)	56 (33.9%)	53 (31.0%)
Widowed	7 (5.1%)	15 (9.1%)	11 (6.4%)
Never married	37 (27.2%)	50 (30.3%)	37 (21.6%)
Domestic partner	4 (2.9%)	2 (1.2%)	6 (3.5%)
**Religious Affiliation ^b^**			
Christian Protestant	44 (40.0%)	52 (39.1%)	51 (39.2%)
Roman Catholic	30 (27.3%)	35 (26.3%)	29 (22.3%)
Jewish	0 (0.0%)	1 (0.8%)	1 (0.8%)
Buddhist	2 (1.8%)	1 (1.5%)	1 (0.8%)
Other	3 (2.7%)	4 (3.0%)	5 (3.8%)
None	31 (28.2%)	39 (29.3%)	43 (33.1%)
CES score [M (SD)]**	22.29 (10.84)	20.56 (11.60)	17.10 (12.65)
PCL total score [M (SD)]	62.67 (11.59)	64.42 (9.93)	64.28 (11.51)

Note. ^a^ Persons in "Other Minority" category predominantly included Asian American, Native American, as well as persons with mixed ethnic backgrounds; ^b^ Question left blank by n=26 in the No Suicide group, n=32 in the Ideation Only group, and n=41 in the Ideation/Attempt group; M = Mean; SD = Standard Deviation; CES = Combat Experiences Scale; PCL = PTSD Clinical Checklist; * p < .05; ** p <.001

MANCOVA with age, gender, ethnicity (non-Caucasian = 0, Caucasian = 1), combat exposure, and PTSD symptom severity as covariates was used to assess differences in spirituality dimensions across the three categories of suicide status (No Suicide, Ideation Only, and Ideation/Attempt). Results indicated a statistically significant effect for the suicide factor, Wilks’ λ = 0.94, F(12, 918.00) = 2.22, p = .009. The Wilks’ λ value found here suggests that the suicide groups have dissimilar means,^58^ highlighting the need for a more indepth examination of differences in spiritual functioning on the basis of Veterans’ history of suicidal thoughts and/or behaviors.

Results for ANCOVAs revealed main effects for three of the spirituality dimensions assessed in the study: forgiveness, F(2, 464) = 6.47, p = .002, negative religious coping, F(2, 464) = 4.79, p = .009, and organizational religiousness, F(2, 464) = 3.61, p = .028.(Table 2) displays the marginal means and homogeneous subgroups (i.e., overlapping 95% confidence intervals) across the spirituality factors for the three groups of suicide risk status.

**Table 2:** Adjusted means and standard errors for spirituality factors (n=472).

	No Suicide (n = 136)		Ideation Only (n = 165)		Ideation/Attempt (n = 171)	
	M	SE	M	SE	M	SE
Daily spiritual experiences	3.75^b^	.121	4.09^a^	.110	3.91^a,b^	.109
Forgiveness	2.35^b^	.061	2.62^a^	.055	2.61^a^	.054
Private religious practices	5.33^a^	.167	5.59^a^	.151	5.42^a^	.150
Positive religious coping	2.69^a^	.077	2.83^a^	.070	2.73^a^	.069
Negative religious coping	3.22^a^	.063	3.05^b^	.057	2.96^c^	.056
Organizational religiousness	6.23^b^	.141	6.74^a^	.128	6.57^b^	.126

Note. M = group mean; SE = standard error of the mean; a, b, and c denote homogenous subsets or statistically equivalent groups of means (i.e., overlapping 95% confidence intervals, group means do not differ significantly). Higher scores indicated lower spirituality for each spirituality factor.

When compared to the No Suicide group, Fisher’s LSD tests revealed that Veterans in both the Ideation Only, p = .001, and Ideation/Attempt, p = .002, groups reported significantly weaker forgiveness at the start of treatment. Veterans in the two suicide groups similarly indicated a greater reliance on negative religious coping compared to their counterparts who did not report any suicidal thoughts and/or behaviors, p < .039. A difference in organizational religiousness also emerged between Veterans with No Suicide and Ideation Only, p = .008, with suicidal thoughts being linked with less involvement in churches or other spiritual communities. Veterans with a history of attempts also had lower scores on the organizational religiousness factor, but this difference failed to reach statistical significance, p = .078. In addition, although we failed to find a statistically significant main effect in the ANCOVA, Veterans in the Ideation Only group reported lesser frequencies of daily spiritual experiences compared to their counterparts in the No Suicide group, p = .034. 

## Discussion

The aim of this study was to examine whether aspects of spiritual functioning were associated with a history of suicidal thoughts and/or behaviors in a sample of Veterans seeking treatment at a residential PTSD care facility. Study hypotheses were largely supported. Firstly, problems with forgiveness and negative religious coping (i.e., indices of spiritual struggle) were uniquely associated with suicidal thoughts and attempts, above and beyond age, gender, or ethnicity, combat exposure, and severity of PTSD symptomatology. Secondly, controlling for these same covariates, involvement in a church or other spiritual community (organizational religiousness) was associated with less suicidal ideation. Thirdly, daily spiritual experiences were inversely associated with suicidal thoughts. Of note, differences failed to emerge between Ideation Only and Ideation/Attempt groups on any of the spirituality factors, suggesting that spirituality was helpful for predicting the presence of suicidal thoughts but not attempts.

The present study represents the first attempt to empirically examine the relationship between spiritual functioning and suicidal thoughts and behavior in a population of Veterans with an increased risk of death by suicide. Thus far, adaptive spiritual functioning has generally been presented as being protective against suicide, mitigating psychological, social, behavioral, and physical risk factors associated with suicidal behavior.^18,59^ These findings reinforced this relationship in part, highlighting the protective potential of daily spiritual experiences and engagement in a church or other type of faith-based community. However, we failed to find that positive religious coping and engaging in spiritual practices was predictive of suicidal thoughts or acts in the presence of age, gender, or ethnicity and PTSD symptom severity.

The results also indicated that maladaptive dimensions (i.e., problems with forgiveness, negative religious coping) of spirituality were associated with suicidality. At present, a limited literature has examined the relationship between forgiveness and suicidal behavior. Self-forgiveness, mediated by depressive symptoms, has been associated with lower levels of suicidal behavior.^60^ Forgiveness of others has been associated with decreased suicidal behavior, independent of depressive symptoms.^60^ Forgiveness of self and others has also been suggested to decrease suicidal behavior through its influence on depressive symptoms, thwarted belongingness, and perceived burdensomeness.^61^ To the best knowledge of the authors, this is the first study to expressly associate problems with forgiveness and negative religious coping (e.g., feeling abandoned by God or a Higher Power, punished for acts of wrongdoing or perceived spiritual weakness, and/or attempting to disengage from God or a Higher Power) with higher levels of suicidal thoughts and suicide attempts. Positive religious coping generally represents strategies for dealing with critical life events in a manner that conserve and protect one’s most cherished life events.^22,23^ In cases of forgiveness problems and negative religious coping, Veterans’ core meaning systems have likely been challenged and they are struggling with the products of a negative transformation in their religious faith or spirituality.

Suicidal behavior is recognized as an immensely complicated clinical construct, reflecting a variety of covariates and considerable heterogeneity among at-risk populations.^62^ While additional research is needed, the present findings suggest that there may be some benefit to focusing on reducing spiritual distress in at-risk Veteran populations as part of organized suicide prevention efforts. For example, religious or spiritual problems are formally recognized as a focus of clinical attention (diagnostic code V62.89).^63^ The findings presented here suggest that such problems may be specifically relevant to suicide prevention efforts.

The present study demonstrated that factors related to spirituality are associated with predicting suicide ideation within the sample population. Considering that ideation is a recognized precursor of more advanced suicidal behavior,^64-67^ spiritual functioning may be most relevant to preventing suicide in the early stages of the suicide trajectory. This highlights the utility of screening for and addressing spiritual distress as part of formal suicide risk assessments and prevention efforts within at-risk Veteran populations. Chaplaincy services are used by some Veterans to deal with spiritual distress and represent a potential adjunctive approach to such efforts.^68^

Findings should be interpreted in the context of several limitations. Firstly, the sample represents a non-randomized sample of convenience, limited to a unique subset of Veterans dealing with PTSD who sought treatment at a single residential care facility. As such, it is reasonable to assume that this group may have been dealing with a more severe and/or chronic form of PTSD for which otherwise conservative treatment strategies (e.g., routine physician visits, outpatient treatment) had proven unsatisfactory. The findings cannot therefore be generalized to all Veterans or civilians dealing with PTSD, nor to the wider Veteran population. As a cross-sectional study, no causal inferences can be made about the relationship between spiritual functioning and suicidal thoughts or behaviors. 

In summary, the present study examined the relationship between spiritual functioning and suicidal thoughts and behaviors in a population of Veterans recognized as being at increased risk of suicide. The findings highlight several avenues for future research. Among others, it would be prudent to examine this relationship in other groups recognized as being at increased-risk of suicide, not limited only to Veteran populations. This could not only help identify differences in spiritual functioning across different at-risk populations, but also help identify any variability in risk based on spiritual functioning. In turn, such research may help inform the development of interventions aimed at applying spiritual functioning to mitigate the risk of suicide in Veteran populations. Longitudinal investigations, in particular, that assess whether spiritual wellbeing is a protective factor for suicide are needed. To the extent that such factors are protective, work is needed to develop, test and incorporate interventions that support spiritual well-being as a component of suicide prevention strategies. 

## References

[1] Kemp JE, Bossarte R. Suicide data report, 2012. Washington, DC: Department of Veterans Affairs, 2013.

[2] Centers for Disease Control and Prevention. National violent death reporting system (NVRDS). 2014, 8 December, http://wisqars.cdc.gov:8080/nvdrs/nvdrsDisplay.jsp, accessed 6 January 2015.

[3] US Department of Veterans Affairs. Implementing VHA’s mental health strategic plan initiatives for suicide prevention. Report no. 06-03706-126. Washington, DC: VA Office of the Inspector General, 2007.

[4] Kemp JE. Suicide Rates in VHA patients through 2011 with comparisons with other Americans and other Veterans through 2010. 2014, 9 January, http://www.mentalhealth.va.gov/docs/Suicide_Data_Report_Update_January_2014.pdf, accessed 6 January 2015.

[5] Bell JB, Nye EC (2007). Specific symptoms predict suicidal ideation in Vietnam combat Veterans with chronic post-traumatic stress disorder. Mil Med.

[6] Hendin H, Haas AP (1991). Suicide and guilt as manifestations of PTSD in Vietnam combat Veterans. Am J Psychiatry.

[7] Pompili M, Sher L, Serafini G, Forte A, Innamorati M, Dominici G (2013). Posttraumatic stress disorder and suicide risk among Veterans: a literature review. J Nerv Ment Dis.

[8] Rozanov V, Carli V (2012). Suicide among war Veterans. Int J Environ Res Public Health.

[9] Sher L, Braquehais MD, Casas M (2012). Posttraumatic stress disorder, depression, and suicide in Veterans. Cleve Clin J Med.

[10] Department of Veterans Affairs. Veterans Posttraumatic Stress Disorder (PTSD). 2014, 10 November, http://www.va.gov/opa/issues/ptsd.asp, accessed 6 January 2015.

[11] Kessler RC, Sonnega A, Bromet E, Hughes M, Nelson CB (1995). Posttraumatic stress disorder in the National Comorbidity Survey. Arch Gen Psychiat.

[12] Kang HK, Natelson BH, Mahan CM, Lee KY, Murphy FM (2003). Post-traumatic stress disorder and chronic fatigue syndrome-like illness among Gulf War Veterans: a population-based survey of 30,000 Veterans. Am J Epidemiol.

[13] Tanielian T, Jaycox LH. Invisible wounds of war: Psychological and cognitive injuries, their consequences, and services to assist recovery. Santa Monica, CA: RAND Corporation, 2008.

[14] Kulka RA, Schlenger WE, Fairbanks JA, Hough RL, Jordan BK, Marmar CR, et al. Trauma and the Vietnam war generation: Report of findings from the National Vietnam Veterans Readjustment Study. New York, NY: Brunner/Mazel, 1990.

[15] Schlenger WE, Kulka RA, Fairbank JA, Hough RL, Jordan BK, Marmar CR (1992). The prevalence of post-traumatic stress disorder in the Vietnam generation: a multimethod, multisource assessment of psychiatric disorder. J Trauma Stress.

[16] Colucci E, Martin G (2008). Religion and spirituality along the suicidal path. Suicide Life Threat Behav.

[17] Kopacz MS, Silver E, Bossarte RM (2014). A position article for applying spirituality to suicide prevention. Journal of Spirituality in Mental Health.

[18] Koenig HG, King DE, Carson VB. Handbook of religion and health. New York:Oxford University Press, 2012.

[19] Fetzer Institute, National Institute on Aging Working Group. Multidimensional measurement of religiousness, spirituality for use in health research. A report of a national working group supported by the Fetzer Institute in collaboration with the National Institute on Aging. Kalamazoo, MI: Fetzer Institute, 1999.

[20] Cole B, Benore E, Pargament K (2004). Spirituality and coping with trauma. In: Sorajjakool S, Lamberton H (eds): Spirituality, health and wholeness: An introductory guide for health care professionals. New York: Haworth Press,.

[21] Ellison CG. Religion, the life stress paradigm, and the study of depression. In: Levin JS (ed): Religion in aging and health. Theoretical foundations and methodological frontiers. Thousand Oaks, CA: A SAGE publication, 1994:78-121.

[22] Pargament K. The psychology of religion and coping: theory, research, practice. New York, NY: The Guilford Press, 1997.

[23] Pargament KI, van Haitsma KS, Ensing DS. Religion and coping. In: Kimble MA, McFadden SH, Ellor JW, Seeber JJ (eds): Aging, spirituality and religion: a handbook. Minneapolis, MN: Fortress Press, 1995:47-67.

[24] Currier JM, Drescher KD, Harris JI (2014). Spiritual functioning among Veterans seeking residential treatment for PTSD: a matched control group study. Spiritual Clin Pract.

[25] Currier JM, Holland JM, Drescher KD (2015). Spirituality factors in the prediction of outcomes of PTSD treatment for U.S. military Veterans. J Trauma Stress.

[26] Fontana A, Rosenheck R (2004). Trauma, change in strength of religious faith, and mental health service use among Veterans treated for PTSD. J Nerv Ment Dis.

[27] Currier JM, Mallot J, Martinez TE, Sandy C, Neimeyer RA (2013). Bereavement, religion, and posttraumatic growth: A matched control group investigation. Psycholog Relig Spiritual.

[28] Fontana A, Rosenheck R (2005). The role of loss of meaning in the pursuit of treatment for posttraumatic stress disorder. J Trauma Stress.

[29] Chang BH, Stein NR, Trevino K, Stewart M, Hendricks A, Skarf LM (2012). Spiritual needs and spiritual care for Veterans at end of life and their families. Am J Hosp Palliat Care.

[30] House Committee on Veterans’ Affairs. Building bridges between VA and community organizations to support Veterans and families. 2012, 27 February, http://Veterans.house.gov/witnesstestimony/m-david-rudd-phd-abpp-2, accessed 6 January 2015.

[31] LaPierre LL (1994). The spirituality and religiosity of Veterans. J Health Care Chaplain.

[32] Pew Social and Demographic Trends. The military-civilian gap, war and sacrifice in the post-9/11 era. Washington, DC: Pew Research Center, 2011.

[33] Greenawalt DS, Tsan JY, Kimbrel NA, Meyer EC, Kruse MI, Tharp DF (2011). Mental health treatment involvement and religious coping among African American, Hispanic, and white Veterans of the wars of Iraq and Afghanistan. Depress Res Treat.

[34] Goertz C, Marriott BP, Finch MD, Bray RM, Williams TV, Hourani LL (2013). Military report more complementary and alternative medicine use than civilians. J Altern Complement Med.

[35] Mihaljević S, Aukst-Margetić B, Vuksan-Ćusa B, Koić E, Milošević M (2012). Hopelessness, suicidality and religious coping in croatian war Veterans with PTSD. Psychiatr Danub.

[36] Kopacz MS (2014). The spiritual health of Veterans with a history of suicide ideation. Health Psychol Behav Med.

[37] The Assessment and Management of Risk for Suicide Working Group. VA/DoD clinical practice guideline for assessment and management of patients at risk for suicide. Washington, DC: Department of Veterans Affairs and Department of Defense, 2013.

[38] Department of Health and Human Services, Office of the Surgeon General, & National Action Alliance for Suicide Prevention. 2012 national strategy for suicide prevention: goals and objectives for action. Washington, DC: Department of Health and Human Services, 2012. 23136686

[39] Simon RI. Preventing patient suicide: Clinical assessment and management. Arlington, VA: American Psychiatric Publishing, 2011.

[40] World Health Organization. Public health action for the prevention of suicide: a framework. Geneva, Switzerland: World Health Organization, 2012.

[41] Currier JM, Holland JM, Drescher KD (2014). Residential treatment for combat-related posttraumatic stress disorder: Identifying trajectories of change and predictors of treatment response. PLoS One.

[42] Idler EL, Musick MA, Ellison CG, George LK, Krause N, Ory MG (2003). Measuring multiple dimensions of religion and spirituality for health research. Conceptual background and findings from the 1998 General Social Survey. Res Aging.

[43] Underwood LG, Teresi JA (2002). The daily spiritual experience scale: development, theoretical description, reliability, exploratory factor analysis, and preliminary construct validity using health-related data. Ann Behav Med.

[44] Underwood LG (2011). The daily spiritual experience scale: overview and results.. Religions.

[45] Mauger PA, Perry JE, Freeman T, Grove DC, McBride AG, McKinney KE (1992). The measurement of forgiveness: preliminary research. J Psychol Christ.

[46] Pargament KI, Koenig HG, Perez LM (2000). The many methods of religious coping: development and initial validation of the RCOPE. J Clin Psychol.

[47] Pargament KI, Zinnbauer BJ, Scott AB, Butter EM, Zerowin J, Stanik P (1998). Red flags and religious coping: identifying some religious warning signs among people in crisis. J Clin Psychol.

[48] Strawbridge WJ, Cohen RD, Shema SJ, Kaplan GA (1997). Frequent attendance at religious services and mortality over 28 years. Am J Public Health.

[49] Blanchard EB, Jones-Alexander J, Buckley TC, Forneris CA (1996). Psychometric properties of the PTSD Checklist (PCL).. Behav Res Ther.

[50] Weathers FW, Ford J. Psychometric properties of the PTSD checklist (PCL-C, PCL-S, PCL-M, PCL-PR). In: Stamm BH (ed): Measurement of stress, trauma, and adaptation. Lutherville, MD: Sidran Press, 1996:250-2.

[51] Weathers FW, Litz BT, Herman DS, Huska JA, Keane TM. The PTSD Checklist (PCL): reliability, validity, and diagnostic utility. Paper presented at the 9th Annual Conference of the ISTSS. San Antonio: TX, 1993.

[52] Keane T, Fairbank J, Caddell J, Zimmering R, Taylor K, Mora C (1989). Clinical evaluation of a measure to assess combat exposure. Psychol Assess.

[53] Pietrzak RH, Johnson DC, Goldstein MB, Malley JC, Southwick SM (2009). Psychological resilience and postdeployment social support protect against traumatic stress and depressive symptoms in soldiers returning from Operations Enduring Freedom and Iraqi Freedom. Depress Anxiety.

[54] Grissom RJ, Kim JJ. Effect sizes for research: Univariate and multivariate applications. New York, NY: Taylor & Francis Group, 2012.

[55] Raykov T, Marcoulides GA. Introduction to applied multivariate analysis. New York, NY: Taylor & Francis Group, 2008.

[56] Ott RL, Longnecker M. An introduction to statistical methods and data analysis, sixth edition. Belmont, CA: Books/Cole, Cengage Learning, 2010.

[57] Howell DC. Fundamental statistics for the behavioral sciences, seventh edition. Belmont, CA: Books/Cole, Cengage Learning, 2011.

[58] Foster J, Barkus E, Yavorsky C. Understanding and using advanced statistics. Thousand Oaks, CA: Sage Publication, 2006.

[59] Brown DR, Carney JS, Parrish MS, Klem JL (2013). Assessing spirituality: the relationship between spirituality and mental health. Journal of Spirituality in Mental Health.

[60] Hirsch JK, Webb JR, Jeglic EL (2011). Forgiveness, depression, and suicidal behavior among a diverse sample of college students. J Clin Psychol.

[62] Nsamenang SA, Webb JR, Cukrowicz KC, Hirsch JK (2013). Depressive symptoms and interpersonal needs as mediators of forgiveness and suicidal behavior among rural primary care patients. J Affect Disord.

[62] Knox KL, Bossarte RM. Suicide prevention for Veterans and active duty personnel. Am J Public Health. 2012 Mar;102 Supple 1:S8-9. 10.2105/AJPH.2011.300593 PMC3496460 22390608

[63] American Psychiatric Association. Diagnostic and statistical manual of mental disorders, fourth edition. Washington, DC: American Psychiatric Association, 1994.

[64] Gliatto MF, Rai AK (1999). Evaluation and treatment of patients with suicidal ideation. Am Fam Physician.

[65] Hall RC, Platt DE, Hall RC (1999). Suicide risk assessment: a review of risk factors for suicide in 100 patients who made severe suicide attempts. Evaluation of suicide risk in a time of managed care. Psychosomatics.

[66] Wilcox HC, Arria AM, Caldeira KM, Vincent KB, Pinchevsky GM, O’Grady KE (2010). Prevalence and predictors of persistent suicide ideation, plans, and attempts during college. J Affect Disord.

[67] Nock MK, Borges G, Bromet EJ, Alonso J, Angermeyer M, Beautrais A (2008). Cross-national prevalence and risk factors for suicidal ideation, plans and attempts. Br J Psychiatry.

[68] Kopacz MS, O’Reilly LM, van Inwagen CC, Bleck-Doran TL, Smith WD, Cornell N (2014). Understanding the role of chaplains in Veteran suicide prevention efforts – a discussion paper. Sage Open.

